# TZDs and Bone: A Review of the Recent Clinical Evidence

**DOI:** 10.1155/2008/297893

**Published:** 2008-09-08

**Authors:** Ann V. Schwartz

**Affiliations:** Department of Epidemiology and Biostatistics, University of California San Francisco, 185 Berry Street, Suite 5700, San Francisco, CA 94107, USA

## Abstract

Over the past two years, evidence has emerged that the currently available thiazolidinediones (TZDs), rosiglitazone, and pioglitazone have negative skeletal consequences, at least in women, which are clinically important. Increased fracture risk in women, but not men, was reported for both TZDs, based on analyses of adverse event reports from clinical trials. In short-term clinical trials in women, both TZDs caused more rapid bone loss. In these trials, changes in bone turnover markers suggest a pattern of reduced bone formation without a change in resorption. Although limited, these results support the hypothesis based on rodent and in vitro models that reduced bone formation resulting from activation of peroxisome proliferator-activated receptor-*γ* (PPAR*γ*) is a central mechanism for TZDs' effect on bone. Research is needed to better understand the mechanisms of bone loss with TZDs, to identify factors that influence susceptibility to TZD-induced osteoporosis, and to test treatments for its prevention.

## 1. INTRODUCTION

Recent reports have substantially advanced our knowledge of the clinical effects of TZDs on skeletal health. In early 2006, research into the skeletal effects in humans of rosiglitazone and pioglitazone, the currently prescribed TZDs, was limited to observational studies [1]. Although a body of evidence had developed from rodent and in vitro studies that these two TZDs cause bone loss, it was not known if these compounds had a similar effect in humans. Since then, rosiglitazone and piogltiazone were each linked to increased fracture risk among diabetic women, based on adverse event reports in clinical trials. And, in women, short-term clinical trials demonstrated substantial bone loss with both TZDs. Pioglitazone and rosiglitazone are widely used to treat diabetes, and better knowledge of their skeletal effects is crucial to guide clinical decisions. At the same time, because TZDs are ligands of PPAR*γ*, a better understanding of their skeletal effects will help to clarify the role of PPAR*γ* in bone metabolism and potentially shed light on the mechanisms of age-related bone loss. This review considers the recent clinical evidence regarding TZDs and skeletal health and discusses outstanding issues that warrant further research.

## 2. ROSIGLITAZONE AND FRACTURE RISK

Evidence that RSG increases fracture risk emerged with the results of the ADOPT trial published in 2006 [2]. A postproof note in the main report from the trial indicated increased fracture risk in women, but not men, enrolled in the trial. Since then, the fracture results have been published separately and in more detail [3]. ADOPT was designed to assess time to monotherapy failure for RSG compared to metformin and to a sulfonylurea, glyburide. The trial had three arms, corresponding to the three different treatments, and enrolled a total of 2511 men and 1840 women who were followed for a median of 4.0 years. The average age was 57 years. By self-report, 77% of women were postmenopausal. Participants were recently diagnosed with diabetes (<3 years), were drug naïve for hypoglycemic medications, and had an average A1C of about 7.4%.

Fractures, identified through adverse event reports, were specifically reviewed after the conclusion of the trial. Based on time to first fracture, the investigators found an increased risk among women in the RSG arm of 1.81 (95% CI: 1.17, 2.80) compared to metformin, and 2.13 (1.30, 3.51) compared to glyburide. The risk for men was not increased compared with either metformin (RH 1.18; 95% CI: 0.72, 1.96) or glyburide (RH 1.08: 95% CI: 0.65, 1.79).

In women, risk was increased for both upper and lower limb fractures. Rate ratios calculated from fracture rates reported for ADOPT showed the largest increases in relative risk for foot (RR = 3.3), hand (RR = 2.6), and proximal humerus (RR > 8) fractures (see Table 1). There was no increased risk identified for clinical spine or hip fractures, but the numbers of these fractures, 3 clinical spine and 4 hip fractures among all women, were too small to draw firm conclusions. The small number of hip and spine fractures in the ADOPT population (average age 57 years) is not surprising since the rate of these fractures tends to be relatively low until after age 65.

**Figure 1:**
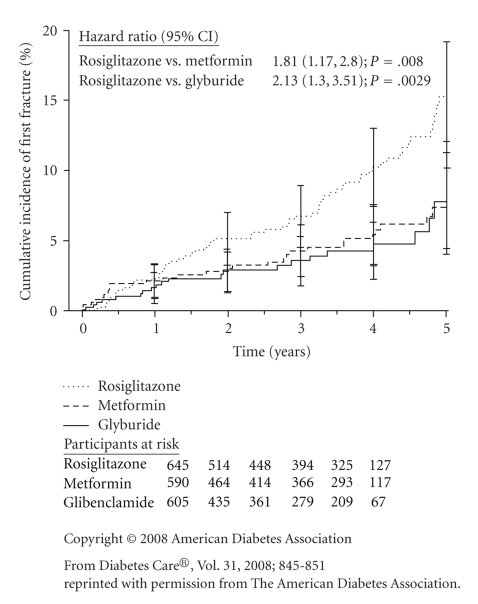
Kaplan-Meier estimates of the cumulative incidence of fractures at five years in women enrolled in ADOPT [3]. Bars represent 95% confidence intervals.

For women, an examination of the survival curves from the ADOPT trial (see Figure 1) suggests that the increased risk of fracture with RSG is evident after about one year of treatment. In separate trials, discussed below, bone loss could be identified among women treated with RSG after only a few months of treatment. However, the ADOPT results suggest that bone loss with RSG does not make a noticeable difference in fracture risk until after about 12 months of treatment.

Self-reported menopausal status and baseline use of estrogen-containing hormones were available for women enrolled in ADOPT. As expected, premenopausal women had a lower rate of fracture than postmenopausal women, but both groups had an approximate doubling of fracture risk with RSG treatment. Menopausal status did not appear to substantially modify the effects of RSG on fracture. About 20% of women reported use of an estrogen-containing hormone at baseline. The effect of RSG on fracture risk did not appear to differ between those who did or did not report estrogen use.

It is possible, though not established, that poor glycemic control increases fracture risk [6]. However, this would not explain the ADOPT results as those in the RSG arm maintained glycemic control on monotherapy longer than those in the metformin or glyburide arms.

## 3. PIOGLITAZONE AND FRACTURE RISK

With the published report of increased fracture risk in the RSG arm of ADOPT, Takeda Pharmaceuticals, IL, USA the manufacturer of pioglitazone, reviewed their clinical trial databases and, in a letter to health care providers in 2007, reported an increased fracture risk with pioglitazone treatment in women, but not men [7]. The databases included 24 000 years of followup for over 8100 patients treated with pioglitazone and over 7400 patients in the comparison group. In these trials, the maximum duration of pioglitazone use was only 3.5 years. The magnitude of the increased risk reported for all clinical fractures was similar to the ADOPT results with a fracture rate of 1.9 per 100 person years in those using pioglitazone compared with a rate of 1.1 per 100 person years in those using placebo or an active comparator drug. The relative risk for men was not reported but was stated to be not statistically significant. Data on specific fracture sites was not provided although the letter stated that most of the fractures occurred in the distal upper limb or distal lower limb.

## 4. TZDs AND BONE LOSS

In 2007, Grey et al. reported the results of a 14-week randomized clinical trial comparing RSG (8 mg/day) with placebo in 50 postmenopausal women, average age 67 years, who did not have diabetes or osteoporosis [8]. The trial found modest reductions in two markers of bone formation. Procollagen type-I *N*-terminal propeptide was reduced by 13% (*P* = .004) and osteocalcin by 10% (*P* = .04) in the RSG arm compared with placebo. In contrast, the bone resorption marker, serum *β*-C-terminal telopeptide (S-CTX) of type I collagen, was stable in the RSG arm and did not differ significantly from placebo (*P* = .9). Substantial bone loss was reported at the total hip with RSG treatment. Women in the RSG group lost bone density (BMD) more rapidly at the total hip (−1.9% RSG versus −0.2% placebo, *P* = .003). For the total spine, bone loss was more rapid in the RSG arm but the difference was not statistically significant (−1.2% RSG versus −0.2% placebo, *P* = .13).

In a randomized, controlled, but unblinded trial, a lower dose of RSG (4 mg/day) for 12 weeks was compared with diet treatment alone in obese postmenopausal women with newly diagnosed diabetes [9]. Bone-specific alkaline phosphatase, a bone formation marker, was decreased in the RSG arm (−21.5%) compared with diet only (−4.1%) (*P* < .05). Osteocalcin was decreased similarly in both arms (RSG −20%; diet only −17.6%) while urine deoxypyridinoline (DPD), a resorption marker, was not increased in the RSG arm (3%) compared with the diet only arm (17%).

The short-term effects of pioglitazone (30 mg/day) on bone density and markers have been tested in a 16-week randomized placebo-controlled trial among 30 premenopausal women with polycystic ovary syndrome (PCOS) [10]. BMD was reduced compared with placebo at the lumbar spine (−1.14% versus 0.00%), total hip (−0.18% versus 1.35%), and femoral neck (−1.45% versus 0.87%) (all *P* < .05). The magnitude of loss in the PIO group at the spine and femoral neck is similar to BMD losses reported with RSG use over 14 weeks in postmenopausal women [8]. Alkaline phosphatase, a marker of bone formation, was decreased in the PIO group compared to placebo but osteocalcin was not. Changes in the marker of bone resorption, S-CTX, were also not significantly different across treatment groups. The treated group experienced a significant decrease in fasting insulin compared to placebo. Since insulin may be anabolic for bone, this may have contributed to the bone loss observed with PIO although the authors reported that the changes in BMD and the changes in insulin were not significantly correlated. Estradiol and testosterone levels were not significantly altered in the PIO group.

Two observational studies have reported results for TZDs and changes in BMD or markers. The first clinical study to report increased bone loss with TZD use, combining troglitazone, rosiglitazone, and pioglitazone, was based on the Health, Aging, and Body Composition longitudinal observational study of older adults [11]. The cohort included 666 diabetic participants with an average age of 73 years. Of these, 69 participants reported any TZD use during four years of followup. Increased bone loss was found in diabetic women but not men. After controlling for potential confounders, the additional bone loss attributed to TZD use in women was −1.23% (95% CI: −2.06%, −0.40%) per year at the lumbar spine, −0.61% (−1.02%, −0.21%) per year for whole body, and −0.49% (−1.04%, 0.07%) for total hip. These estimates of increased bone loss are substantially lower than those reported by Grey et al. [8] for the trial of RSG use and by Glintborg et al. [10] for the trial of PIO use. The additional bone loss of 1.5–1.7% at the total hip over 14–16 weeks in these two trials, if sustained, would result in additional bone loss of 5-6% annually. While the observational study by Schwartz et al. may have underestimated the degree of bone loss associated with TZD use, it seems unlikely that bone loss of 6% per year is occurring with TZD use. Instead, there may be an initial period of more rapid bone loss, followed by continued loss at a lower rate, similar to the effect of glucocorticoids [12].

Although Schwartz et al. reported no increased bone loss with TZD use in diabetic men, Yaturu et al., in an observational study of 160 older diabetic men (average age 68 years), did report that RSG use (*N* = 32) was associated with increased bone loss of −1.05% per year at the total hip, −1.02% at the femoral neck, and −1.24% at the spine (all *P* < .03) [13]. However, the study did not have sufficient power to control for potential confounders such as A1C level, use of other medications, or diabetic complications.

### 4.1. Rodent and in vitro models

Results of rodent and in vitro models provided the first evidence that RSG and PIO cause bone loss. RSG has been more extensively studied in these models but both compounds are associated with bone loss in rodents [14, 15]. These findings have been reviewed previously [16, 17] and will not be discussed in depth here. However, a few points are worth noting as particularly relevant to future research in humans. In general, these models indicate a negative effect on osteoblast differentiation and activity with a decrease in bone formation. However, in a few reports, TZDs were associated with increased resorption. Notably, this occurred in ovariectomized rats [18] and in aged mice [19]. Sottile et al. reported that ovariectomized rats experienced bone loss with RSG, but intact female rats did not, and that the bone loss was characterized by increased resorption [18]. This suggests an interaction between RSG and estrogen levels that needs to be assessed in human studies. The results from Lazarenko et al. comparing the effects of RSG in young, adult, and aged mice suggest that the mechanism of action may be different in the aged mice [19]. In young and adult mice, bone loss with RSG treatment was driven by reduced formation while in older mice RSG treatment resulted in increased resorption. These results need to be explored in human studies as they would suggest different approaches to treatment for the prevention of TZD-induced osteoporosis.

## 5. FUTURE DIRECTIONS FOR CLINICAL RESEARCH

Substantial evidence has now emerged that RSG and PIO have clinically important negative skeletal effects. Increased fracture risk in women, but not men, has been reported for both RSG and PIO. Although this increased fracture risk was identified in the context of clinical trials, the fractures were identified through adverse event reports and were not a planned outcome of the trials. It is possible for adverse event results in a clinical trial to give a signal that is statistically significant due to chance rather than to an actual effect of the intervention. However, the fracture effect is consistent with two clinical trials demonstrating bone loss with RSG and PIO. And, the increased fracture risk and bone loss are consistent with the results of rodent and in vitro models. The combination of these studies provides a compelling argument that, in women, the two currently prescribed TZDs cause higher fracture risk due to bone loss.

Given this growing evidence of increased fracture risk and bone loss with TZD use, further exploration of the skeletal effects of TZDs is crucial to inform efforts to prevent TZD-induced osteoporosis and, more generally, to delineate the role of PPAR*γ* in bone metabolism. Some of the key questions for clinical research are identified and discussed below.

### 5.1. What groups are at higher risk?

To inform clinical decisions and to better understand the mechanism of TZDs effects on the skeleton, it is important to ascertain if there are groups that are particularly vulnerable, or groups that are not susceptible, to increased fracture risk with TZD use. So far, the negative skeletal effects seem to be more important for women than for men, but results are not conclusive. Among women, menopausal status does not appear to modify the effect of RSG on the skeleton. The ADOPT results indicate that increased fracture risk extends to those who are premenopausal as well as postmenopausal. Both premenopausal [10] and postmenopausal [8] women have been shown to lose bone with TZD treatment.

A possible explanation for the lack of effect on the skeleton in men is the higher estrogen levels found in older men compared with older women. In a rat model, ovariectomized, but not intact, females had bone loss with RSG treatment, suggesting a protective effect from higher estrogen levels [18]. However, clinical results to date indicate that TZDs cause increased bone loss and fracture risk in pre- as well as postmenopausal women. Further research with measurements of endogenous estrogen levels could clarify whether there is an interaction between estrogen levels and TZD use.

### 5.2. What happens to bone density and turnover after 3-4 months of treatment?

The randomized trials with RSG and PIO have reported on treatment for 14–16 weeks. In both trials, the additional bone loss in the treated group was substantial, equivalent to a loss of 5-6% over a year, but it seems unlikely that this rate of loss is being sustained over longer treatment periods. Observational studies suggest increased loss of about 0.5–1% each year. Steroid treatment appears to cause initial rapid bone loss followed by continued loss but at a lower rate; the TZDs may present a similar pattern [12]. However, trials of longer duration are needed to assess the degree of loss over several years.

### 5.3. Effect on resorption as well as formation?

One of the key questions regarding the mechanism of action of the TZDs is whether bone resorption and formation, or only one, are affected. The clinical evidence to date, based on bone turnover markers, points to a reduction in bone formation without a change in bone resorption. However, these results are based on only three studies that included bone marker results [8–10]. Rodent models have generally shown reduced bone formation but, in aged mice and in ovariectomized rats, bone resorption is increased. Whether bone resorption is similarly increased with older age or with very low endogenous estrogen levels in human studies has not been fully explored.

### 5.4. Do effects on cortical and trabecular bone differ?

The increased fracture risk observed in the bones of the extremities, that have a relatively high proportion of cortical bone, suggests a negative impact on cortical bone. This pattern is distinct from glucocorticoids which have a particularly strong effect on trabecular bone and the risk of vertebral fracture [12]. Studies using imaging techniques that can separate these two compartments, such as high resolution computed tomography, could clarify whether the effects of TZDs differ for cortical and trabecular bones.

### 5.5. Marrow adiposity

In most reports from rodent models, increased marrow adiposity accompanies bone loss with RSG treatment. Further investigation of this phenomenon has suggested that activation of PPAR*γ* with RSG increases lineage allocation of stem cells towards adipocytes at the expense of osteoblasts in the marrow. To date, human studies have not measured bone marrow adiposity. Knowledge of the effect of TZDs on bone marrow fat would increase our understanding of the mechanisms underlying bone loss and fracture risk in humans with TZD use. In addition, an increase in bone marrow fat may cause an artificial decrease in BMD measured by DXA [20]. If marrow fat is increased, the degree of bone loss with TZD use may be overestimated by DXA measurements.

### 5.6. Effective treatment for TZD-induced osteoporosis

There are no studies to date on treatments that might prevent TZD-induced bone loss. Although the bisphosphonates mainly target bone resorption, the general reduction in bone turnover may be efficacious in preventing bone loss with TZD treatment. The bisphosphonates are successfully used for prevention of osteoporosis with corticosteroid treatment, also characterized by reduced bone formation [21]. However, TZDs have specific effects on bone, and bisphosphonate use should be explicitly tested to determine efficacy in this situation. Treatments that increase bone formation, currently limited to parathyroid hormone (PTH) and strontium ralenate, could theoretically prevent TZD-induced bone loss. PTH has been shown to prevent bone loss with glucocorticoid therapy [22], but neither treatment has been tested in relation to TZDs.

## 6. CONCLUSION

Research over the past two years has provided new clinical evidence that the currently prescribed TZDs increase fracture risk and bone loss, at least in women. Combined with the findings from rodent and in vitro models, these clinical results suggest that activation of PPAR*γ* can play a role in bone loss. With the widespread use of TZDs as a diabetes treatment, further research is needed to delineate the groups that are most susceptible to TZD-induced osteoporosis, to determine the rate of bone loss with TZD treatment beyond 16 weeks, to assess the effects of TZDs on marrow adiposity, cortical and trabecular bones, and to identify treatments to prevent TZD-induced fracture risk. Addressing these questions will advance our ability to prevent TZD-induced osteoporosis and will provide a better understanding of the role of PPAR*γ* activation in bone metabolism.

## Figures and Tables

**Table 1:** Fracture rates comparing rosiglitazone with metformin or glyburide in ADOPT study. Table adapted from a Letter to Health Care Providers issued by GSK [4].

	Rosiglitazone		Metformin or glyburide		Relative rate (95% CI)	
*Women*						
Total followup (P-Y)	2187.20		3578.80			

Fracture site	*N*	Rate/100PY	*N*	Rate/100PY	RR	(95% CI)

Lower limb*	36	1.65	26	0.73	2.27	(1.33, 3.91)
Hip	2	0.09	2	0.06	1.64	(0.12, 22.57)
Foot	22	1.01	11	0.31	3.27	(1.52, 7.47)
Upper limb^†^	22	1.01	19	0.53	1.89	(0.98, 3.70)
Hand	8	0.37	5	0.14	2.62	(0.76, 10.17)
Humerus	5	0.23	0	0.00	^‡^	(1.50,^‡^)
Spine	1	0.05	2	0.06	0.82	(0.01, 15.72)
Other	5	0.23	8	0.22	1.02	(0.26, 3.55)

All fractures	64	2.93	55	1.54	1.90	(1.31, 2.78)

*Men*					
Total followup (P-Y)	2766.70		5570.40			

	*N*	Rate/100PY	*N*	Rate/100PY	RR	(95% CI)
Total participants with any fracture	32	1.16	57	1.02	1.13	(0.71, 1.77)

* Hip, foot, ankle, femur, fibula, lower limb (general), patella, tibia. ^†^ Hand, humerus, clavicle, forearm, radius, upper limb (general), wrist. ^‡^ Cannot estimate. No events in the comparison group. Reprinted with permission from [5]
